# Prospective Evaluation of Sarcopenia in Head and Neck Cancer Patients Treated with Radiotherapy or Radiochemotherapy

**DOI:** 10.3390/cancers13040753

**Published:** 2021-02-11

**Authors:** Sébastien Thureau, Lucie Lebret, Justine Lequesne, Marine Cabourg, Simon Dandoy, Céline Gouley, Laureline Lefebvre, Romain Mallet, Sorina-Dana Mihailescu, Cristian Moldovan, Olivier Rigal, Ovidiu Veresezan, Romain Modzewelski, Florian Clatot

**Affiliations:** 1 Department of Radiation Oncology, Henri Becquerel Cancer Center & QuantIF—LITIS [EA (Equipe d’Accueil) 4108], 76038 Rouen, France; 2 Department of Radiation Oncology, Henri Becquerel Cancer Center, 76038 Rouen, France; Lucie.lebret@chb.unicancer.fr (L.L.); simon.dandoy@chb.unicancer.fr (S.D.); laureline.lefebvre@chb.unicancer.fr (L.L.); romain.mallet@chb.unicancer.fr (R.M.); ovidiu.veresezan@chb.unicancer.fr (O.V.); 3 Department of Statistics and Clinical Research Unit, Henri Becquerel Cancer Center, 76038 Rouen, France; justine.lequesne@chb.unicancer.fr (J.L.); Sorina-Dana.Mihailescu@CHB.unicancer.fr (S.-D.M.); 4 Department of Medical Oncology, Henri Becquerel Cancer Center, 76038 Rouen, France; marine.cabourg@chb.unicancer.fr (M.C.); cristian.moldovan@chb.unicancer.FR (C.M.); Olivier.rigal@chb.unicancer.fr (O.R.); florian.clatot@chb.unicancer.fr (F.C.); 5 Department of Supportive Cancer, Henri Becquerel Cancer Center, 76038 Rouen, France; celine.gouley@chb.unicancer.fr; 6 Department of Nuclear Medicine, Henri Becquerel Cancer Center & QuantIF—LITIS [EA (Equipe d’Accueil) 4108—FR CNRS 3638], Faculty of Medicine, University of Rouen, 76130 Rouen, France; romain.modzewelski@chb.uhnicancer.fr

**Keywords:** radiotherapy, radiochemotherapy, sarcopenia, head and neck squamous cell carcinomas

## Abstract

**Simple Summary:**

Radiation therapy (RT) is a key treatment for head and neck squamous cell carcinoma (HNSCC) patients. RT generates acute toxicity and weight loss, especially when combined with chemotherapy. Frail patients (malnourished or with poor performance status) have poor outcomes and increased toxicity when treated by RT. Among non-frail patients, predicting RT outcome is a challenge. Loss of muscle mass, also known as sarcopenia, is associated with poor outcome in HNSCC patients treated by RT and chemotherapy, but the level of evidence remains weak. Conflicting results exist regarding the impact of sarcopenia on acute RT toxicity. This prospective study confirmed that in non-frail HNSCC patients baseline sarcopenia is frequent (37%) and is associated with decreased overall and disease-free survival, but not with acute toxicity. Interestingly, a worse impact of sarcopenia occurred despite optimal nutritional support and even when patients were treated by RT without chemotherapy. Sarcopenia should be regarded as an independent prognostic factor in that setting.

**Abstract:**

*Highlights:* Sarcopenia is frequent in patients treated with radiation therapy (RT) or radiochemotherapy (RTCT) for head and neck squamous cell carcinomas. Sarcopenia is associated with poor disease-free survival and overall survival outcomes. Sarcopenia is not associated with a higher rate of treatment-related toxicity. *Background:* Sarcopenia occurs frequently with the diagnosis of head and neck squamous cell carcinoma (HNSCC). We aimed to assess the impact of sarcopenia on survival among HNSCC patients treated with radiotherapy (RT) or radiochemotherapy (RTCT). *Methods:* Patients treated between 2014 and 2018 by RT or RTCT with curative intent were prospectively included (NCT02900963). Optimal nutritional support follow-up, including weekly consultation with a dietician and an oncologist and daily weight monitoring, was performed. Sarcopenia was determined by measuring the skeletal muscles at the L3 vertebra on the planning CT scan for radiotherapy. For each treatment group (RT or RTCT), we assessed the prognostic value of sarcopenia for disease-free survival (DFS) and overall survival (OS) and its impact on treatment-related toxicity. *Results:* Two hundred forty-three HNSCC patients were included: 116 were treated by RT and 127 were treated by RTCT. Before radiotherapy, eight (3.3%) patients were considered malnourished according to albumin, whereas 88 (36.7%) patients were sarcopenic. Overall, sarcopenia was associated with OS and DFS in a multivariate analysis (HR 1.9 [1.1–3.25] and 1.7 [1.06–2.71], respectively). It was similar for patients treated with RT (HR 2.49 [1.26–4.9] for DFS and 2.24 [1.03–4.86] for OS), whereas for patients treated with RTCT sarcopenia was significantly associated with OS and DFS in univariate analysis only. Sarcopenia was not related to higher treatment-related toxicity. *Conclusions:* Pretherapeutic sarcopenia remains frequent and predicts OS and DFS for non-frail patients treated with curative intent and adequate nutritional support.

## 1. Introduction

Head and neck squamous cell carcinomas (HNSCCs) are serious conditions with often late diagnosis and high mortality; there were 835,000 new cases and 428,000 deaths worldwide in 2018 [1]. However, HNSCC is a heterogeneous disease with clinical and histological prognostic factors that greatly modify outcomes. Most HNSCCs are diagnosed at an advanced stage [2], and despite multimodal treatment prognosis remains poor, with 50% overall survival at five years [3]. Radiation therapy (RT) is a key treatment in HNSCCs. In the case of inoperable locally advanced stages (T3–T4 and/or N+), RT is usually associated with systemic therapy with either cisplatin or cetuximab. In the case of operated tumors, RT is also frequently proposed. The addition of cisplatin to RT (RTCT) is undertaken when histological risk factors for relapse are observed, such as invaded margins or extracapsular spread. Postoperative RTCT is associated with a higher rate of toxicity than RT [4] and, in particular, greater weight loss [5]. Even if RTCT is usually restricted to selected patients—i.e., those without significant cardiovascular comorbidities or malnutrition—most patients receiving RTCT do not complete a full course of treatment, which can impact their outcomes [6]. Unfortunately, a randomized study showed that pretherapeutic aggressive nutritional support does not prevent weight loss in this setting [7]. The identification of other prognostic or modifiable factors before the initiation of RT is thus necessary.

Sarcopenia is associated with low skeletal muscle mass (LSM) and a decrease in muscle function, and it has been intensively studied over the last decade, primarily in the geriatric population. Sarcopenia or LSM has been independently associated with poor outcomes in various cancer situations [8,9] or toxicities [10,11]. The gold standard for determining body composition and LSM is based on a lumbar vertebra 3 (L3) CT scan analysis, but some studies have used an extrapolation from cervical vertebra 3 (C3) CT scan analysis or MRI or a thoracic vertebral 12 (T12), which is more often available in HNSCCs [12,13,14]. Several studies have recently investigated the potential impact of sarcopenia or LSM on HNSCC patient outcomes. Surprisingly, the pretreatment LSM rate varied greatly between studies, from 6.6% among 258 patients [15] to 65% among 216 patients [16]. In addition to the various cut-off values used to define LSM among studies, the baseline characteristics of the included patients have also varied greatly, which might partially explain such differences.

Considered together, published data have reported that pretreatment LSM in HNSCC patients seems to be independently related to poor outcomes in terms of disease-free survival (DFS) (time from inclusion to the recurrence of tumor or death) and overall survival (OS) [15,17,18,19,20]. Nevertheless, nutritional surveys and support during RT-RTCT are heterogeneous and can affect treatment outcomes. To our knowledge, the impact of sarcopenia on DFS and OS in a prospective cohort with optimal nutritional support has not yet been reported. Available data regarding the impact of sarcopenia on treatment-related toxicity are less clear. The largest study reported a higher rate of toxicity when assessed by a physician but not when assessed by patients [18].

In this context, we aimed to evaluate the incidence and impact of baseline L3-defined LSM on toxicities and outcomes in a homogenous cohort of patients treated by RT or RTCT with curative intent; with optimal nutritional support, including weekly consultation with a dietician and an oncologist; and daily weight monitoring. Since patients treated by RT or RTCT have highly different baseline characteristics and treatment-related toxicities, these two populations were evaluated separately.

## 2. Materials and Methods

The NutriNeck (NCT02900963) trial is an observational prospective, unicentric study that was conducted at the Henri Becquerel Centre (Rouen, France) between April 2014 and March 2018. The study was funded by the Departments of Radiation Therapy and Supportive Care. The inclusion criteria were as follows: primary tumor with a pathological diagnosis of HNSCC, no distant metastasis, at least 18 years of age, World Health Organization Performance Status (WHO PS) ≤ 2, and treatment by radiotherapy with curative intent combined or not with surgery and systemic treatment. Patients who had already received enteral nutrition were not included.

The patients were all treated with intensity-modulated radiation therapy (IMRT) or volumetric arc therapy (VMAT) using a simultaneous integrated boost (SIB). Radiotherapy was administered 5 days per week; the dose distribution was defined according to the risk of locoregional dissemination following international recommendations. Two or three dose levels were prescribed: high risk level (66 to 70 Gy), intermediate risk level if needed (equivalent dose 60 Gy), and low risk level (equivalent dose 45 to 50 Gy). Doses were prescribed at the International Commission on Radiation Units and Measurements (ICRU) point. Chemotherapy treatment, when indicated, was 100 mg/m^2^ of cisplatin on D1–D22–D43 from the first day of radiotherapy. Cetuximab could be used in some inoperable patients with contraindications to cisplatin. Cetuximab was administered one week before radiation at a dose of 400 mg/m^2^ and then weekly at a dose of 250 mg/m^2^ during radiotherapy.

A treatment was considered complete if patients had a full planned RT dose delivered without RT interruption of more than 3 days and if all planned cycles of CT or cetuximab were administered without dose reduction for patients in the RTCT group.

Systematic nutritional management was defined. During radiotherapy, the patients were weighted every day and had weekly consultations with 3 different dieticians and 3 radiation oncologists with a standardized evaluation. In the case of a 2 kg decrease from the first day of radiotherapy, a dedicated medical consultation was performed to optimize the nutrient intake and analgesic treatment. Enteral nutrition was proposed in two following situations: no nutrient intake was possible or additional weight loss was observed on subsequent days.

### 2.1. Patients’ Characteristics

The following parameters were collected: gender, age, WHO PS, smoking and alcohol history, height (cm), weight (kg), body mass index (BMI), cancer site, tumor node metastasis stage (TNM) according to the 7th edition of the American Joint Committee on Cancer Staging, Human PapillomaVirus (HPV) status for oropharyngeal cancer, nutritional status (Albumin dosage), treatment modality (surgery, chemotherapy), toxicity (Common Terminology Criteria for Adverse Events: CTCAE 4.0 for mucositis, dermatis, dysphagia, xerostomia, pain, anorexia, nausea, vomiting), and treatment compliance.

### 2.2. CT Scan

**Figure 1:**
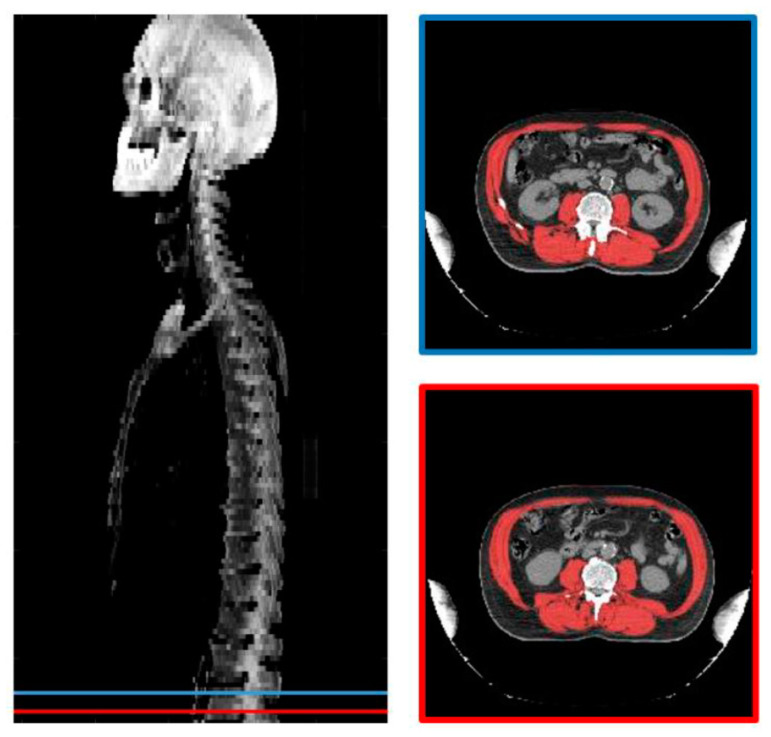
(**Left**) coronal (Maximum Intensity Projection) MIP centered on the vertebral column and the 2 slices selected at the L3 level; (**right**) axial CT slices selected and the segmentation of the skeletal muscles.

All the radiotherapy planning CT scans used for sarcopenia assessment were performed on the same device (Lightspeed Optima CT580, GE, Boston, MA, US) under the same acquisition (120 kV, injected) and reconstruction conditions (Body Filter, 5 mm slice thickness). Sarcopenia was assessed by software running as a plugin on our institutional Picture Archiving and Communication System (PACS, Telemis version 4.7, Telemis SA, Louvain la Neuve, Belgium). The radiation oncologist selected two cross-sectional CT slices at the L3 level separated by one cm. The skeletal muscles were automatically delineated by a deep learning segmentation algorithm. [21] All of the delineations were visually checked and corrected, if necessary, by the radiation oncologist. The L3 skeletal muscles were the psoas, quadratus lumborum, paraspinal, and abdominal wall muscles. The mean of the delineated surfaces on both images was defined as the skeletal muscle L3 area (cm^2^) (Figure 1). The skeletal muscle index was calculated by dividing the skeletal muscle area by the squared height (SMI, cm^2^/m^2^) [22]. Sarcopenia was defined as SMI < 52.4 cm^2^/m^2^ for men and <38.5 cm^2^/m^2^ for women [23].

### 2.3. Statistics

The statistical analysis was conducted using R software, version 3.6.1 [24]. Continuous data were compared using independent samples t tests, and categorical data were compared using the chi-square test or Fisher’s exact test. Median follow-up was calculated using the reverse Kaplan–Meier method. Survival probabilities were estimated using the Kaplan–Meier method, and the log-rank test was performed to evaluate the effects of sarcopenia on survival in the entire cohort regarding the received treatment. Cox models were used to predict disease-free survival (DFS) and overall survival (OS) in the entire cohort, as well as in the RT and RTCT subgroups, according to clinically pertinent and literature-found variables. Covariates for the multivariable Cox proportional hazards regression models were selected using stepwise-forward selection with the condition *p*-value = 0.157 [25]. A two-tailed *p*-value less than 0.05 was considered statistically significant.

## 3. Results

### 3.1. Patient Characteristics

The 243 patients included 187 (77%) males and 56 (23%) females, with a median age of 61 years old (95% CI 56–66). Among them, 81.5% were former or current smokers and 48.6% were chronic alcohol drinkers. The most common tumor site was the oropharynx (*n* = 79, 32.5%), followed by the oral cavity (*n* = 69, 28.4%), larynx (*n* = 39, 16.9%), and hypopharynx (*n* = 39, 16.9%). Seventeen patients (7%) had a cancer of unknown primary (CUP). The most frequent stage was stage IV (*n* = 117, 48.1%), followed by stage III (*n* = 57, 23.5%), stage II (*n* = 47, 19.3%), and stage I (*n* = 19, 7.8%). Among patients with oropharyngeal cancer, p16 positivity was found in 23 patients (29.1%), p16 negativity was found in 30 patients (38%), and p16 status was not available for 26 patients (32.9%). Ninety-seven patients were WHO PS 0 (40.1%), 127 were WHO PS 1 (52.5%), and 18 were WHO PS 2 (7.4%). According to the albumin baseline level, eight patients were undernourished (albumin < 35 g/L). Based on the baseline SMI determined at L3-CT, 88 of 243 patients had sarcopenia (36.7%). Patient characteristics were reported in Table 1.

### 3.2. Patient Characteristics According to Treatment

Overall, 116 patients (47.7%) were treated with RT, preceded by surgery for 89 patients and with radiotherapy alone for 27 patients, while 127 patients (52.3%) were treated by RTCT, preceded by surgery for 63 patients and by exclusive RTCT for 64 patients. Among the 127 patients treated by RTCT, 16 (12.6%) received cetuximab and 111 (87.4%) received cisplatin. The rate of prior surgery was more frequent among patients treated by RT without CT than among patients treated by RTCT (*p* < 0.0001). Patients treated with RTCT were significantly younger and had a more advanced tumor stage than those treated with RT (mean age 59 versus 63.4 years old, *p* = 0.00014; 58.3% stage IV versus 37.1%, *p* < 0.0001, respectively). Smoking history, chronic alcohol consumption, BMI, localization, WHO PS, p16 status, and undernutrition did not differ between the RT and RTCT groups.

The rate of sarcopenic patients was not significantly different between the groups, with 48 patients (41.7%) in the RT group versus 40 patients (32%) in the RTCT group (*p* = 0.12). Patient characteristics according to the sarcopenic status for each treatment group are specified in Table S1.

### 3.3. Treatment Compliance and Toxicity

**Figure 2:**
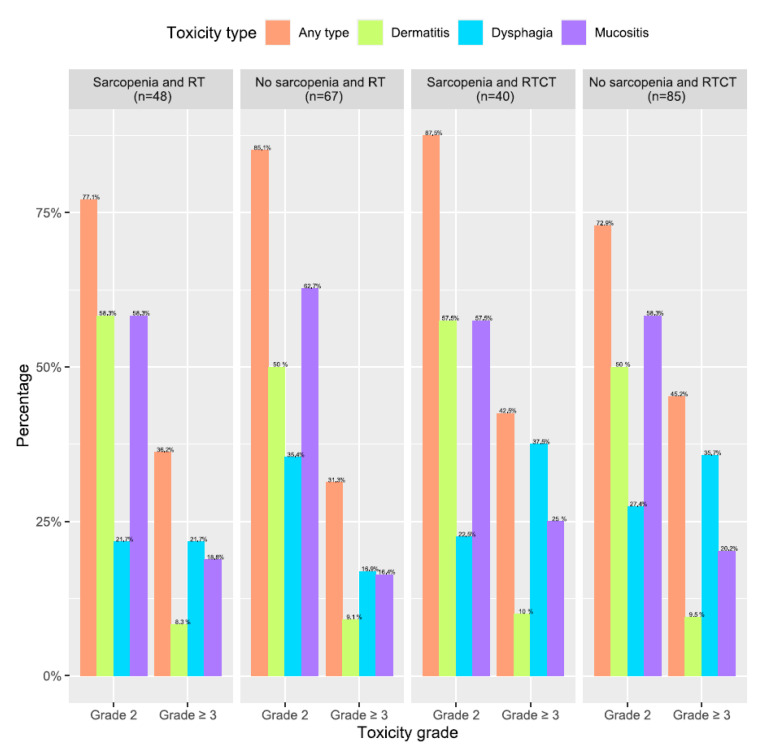
Percentages of patients with grade 2 or grade 3 acute toxicities according to sarcopenic status and treatment. The “any type” column represents the percentage of patients with at least one type of toxicity. The same patient could have several types of grade or 3 toxicities.

Complete treatment was observed in 182 patients (74.9%): 113 of 116 patients in the case of RT (97%) and 69 of 127 (54%) in the case of RTCT (*p* < 0.0001). In the entire cohort, 215 of 243 patients experienced at least one grade 2 toxicity (85%); 103 (47.9%) were treated by RT and 112 (52.1%) were treated by RTCT. Moreover, 95 of 243 (39.9%) patients experienced at least one grade 3 toxicity at the end of treatment; 39 (41.1%) were treated by RT and 56 (58.9%) were treated by RTCT. Overall, there was no significant difference between RTCT and RT regarding the rate of grade 3 toxicity (44.8% vs. 34.5%, respectively, *p* = 0.11). Sarcopenia had no impact on toxicity for mucositis, dermatitis, or dysphagia in either the radiotherapy or chemoradiotherapy group (Table 2 and Figure 2). During treatment, 160 of 243 patients (68%) had a feeding tube indication. Notably, 106 of 127 patients in the RTCT group had an indication for a feeding tube (83.5%). Due to feeding tube refusal, only 108 patients used a feeding tube: 28 (25.9%) in the case of RT and 80 (74.1%) in the case of RTCT (*p* < 0.0001) (Table S1).

### 3.4. Survival Endpoints

**Figure 3:**
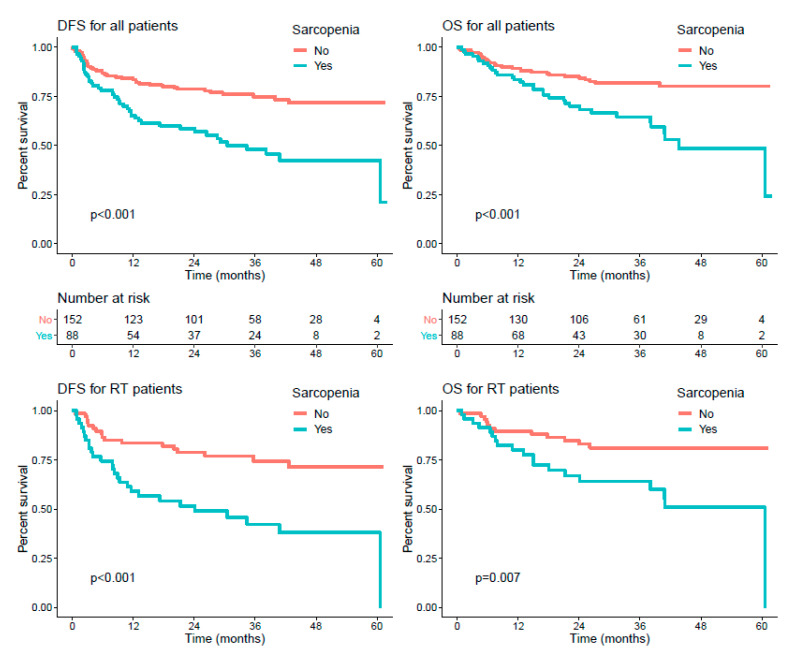

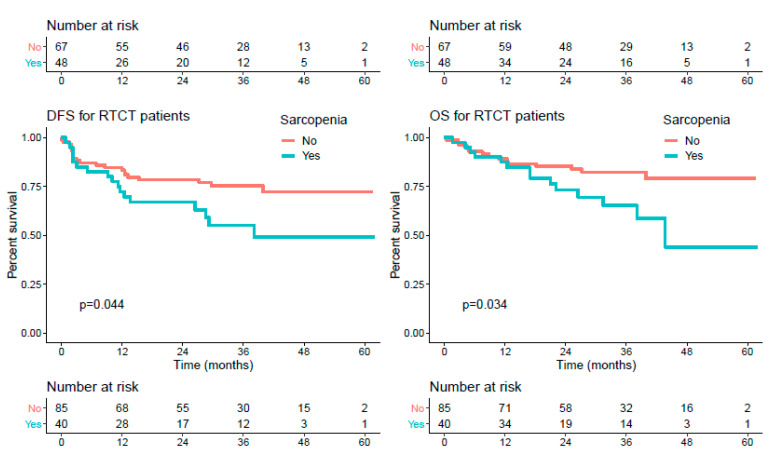
Kaplan–Meier curves of disease-free survival (DFS) and overall survival (OS) for the entire population (**top**) and for patients treated with RT (**middle**) and RTCT (**bottom**).

The median follow-up was 36 (95% CI 33.3–39.7) months. Among the 243 patients, 61 (25.1%) died and 83 (34.2%) progressed. In the whole cohort, univariate analyses showed that WHO PS 2 (versus 0–1), stage III-IV (versus I-II), undernutrition, and sarcopenia were significantly associated with poorer OS. WHO PS, stage III-IV, undernutrition, previous surgery, treatment by RTCT, and sarcopenia were significantly associated with poorer DFS (*p* < 0.05). In multivariate analyses, only WHO PS (HR: 4.26 [2.08–8.73]) and sarcopenia (HR: 1.9 [1.11–3.25]) were significantly associated with OS, while WHO PS (HR: 4.14 [2.19–7.84]), stage (HR: 2.1 [1.11–3.94]), sarcopenia (HR: 1.7 [1.06–2.71]), and treatment by RTCT (HR: 0.57 [0.34–0.96]) remained significantly associated with DFS (Table 3 and Figure 3 top).

When considering treatment subgroups, for patients treated by RT, WHO PS (HR: 9.12 [4.2–19.8]), undernutrition (HR: 3.67 [1.13–11.9]), previous surgery (HR: 0.49 [0.26–0.94]) and sarcopenia (HR: 2.94 [1.6–5.42]) were associated with DFS in univariate models, and WHO PS (HR: 5.9 [2.38–14.61]), stage (HR: 2.27 [1–5.14]), undernutrition (HR: 5 [1.37–18.32]), previous surgery (HR: 0.29 [0.13–0.62]), sarcopenia (HR: 2.49 [1.26–4.9]), and p16 status (HR: 5.04 [1.08–23.64]) were associated with DFS in multivariate models. Similarly, regarding OS, WHO PS (HR: 6.69 [2.73–16.4]), undernutrition (HR: 6.24 [1.88–20.7]) and sarcopenia (HR: 2.71 [1.31–5.59]) were associated with poorer OS in univariate models, and WHO PS (HR: 4.23 [1.45–12.33]), stage (HR: 2.69 [0.94–7.67]), undernutrition (HR: 12.25 [2.93–51.15]), and sarcopenia (HR: 2.24 [1.03–4.86]) were associated with poorer OS in multivariate models (Table 4 and Figure 3 middle).

For patients treated by RTCT, WHO PS (HR: 4.94 [1.91–12.79]), stage (HR: 8.84 [1.21–64.41]), and sarcopenia (HR: 1.93 [1.02–3.67]) were associated with DFS in univariate models, and WHO PS (HR: 4.57 [1.74–11.98]) and stage (HR: 7.49 [1.02–55.13]) were associated with DFS in multivariate models. Regarding OS, WHO PS (HR: 5.15 [1.76–15.1]), stage (HR: 6.58 [0.89–48.4]), and sarcopenia (HR: 2.21 [1.06–4.59]) were associated with univariate models, and WHO PS (HR: 4.84 [1.63–14.36]) and stage (HR: 5.59 [0.75–41.65]) were associated with multivariate models (Table 4 and Figure 3 bottom).

## 4. Discussion

In this study, pretreatment LSM was observed in 88 of the 243 prospectively included patients (36.7%) and was independently associated with poorer DFS and OS. This negative impact of sarcopenia was observed among patients treated by RT or RTCT. In contrast, baseline sarcopenia was not associated with a higher risk of radiation-induced toxicity.

The baseline characteristics of the patients included are in line with the published data in this setting, with mainly locally advanced tumors (stage III-IV). Interestingly, the median BMI was 25 kg/m^2^, eight of 243 patients (3.3%) were considered malnourished at inclusion, and only 18 patients had a baseline WHO PS > 1 (7.4%). In contrast, sarcopenia was observed in 36.7% of the patients, in line with the published data [13,14]. Thus, CT-defined sarcopenia can be frequently identified for patients who are not regarded as frail.

Several large studies have already reported a poorer PFS or OS in the case of sarcopenia [11,13,14,20]. Recently, a systematic review evaluated the impact of CT-defined sarcopenia among HNSCC patients treated with RT [26]. This review emphasized the high heterogeneity of available data in terms of patient selection, sarcopenia definition, or treatment received. Thus, the authors concluded that the overall certainty of the evidence that sarcopenia was associated with reduced OS was low. Based on a prospective cohort using the gold-standard L3-based LSM assessment, our results confirmed that sarcopenia was independently associated with DFS, OS, advanced stages, and low PS status. One of the strengths of our study is that we did not include frail patients with a PS of 3 or more nor patients who had already received enteral nutrition at inclusion. Moreover, patients could have predefined nutritional weekly monitoring during treatment. Thus, the selection bias of frail patients or patients who did not access supportive care was limited. Moreover, in this study the impact of sarcopenia on outcomes was analyzed separately according to treatment group (RT/ RTCT). As expected, the patients treated by RT as part of multimodal treatment had lower-stage disease and were slightly older than the patients treated by RTCT. However, there was no difference between the RT and RTCT groups when considering PS status, BMI, or sarcopenia. In both groups, WHO PS 2 vs. WHO PS 0–1 and stage III–IV disease vs. stage I-II disease were independently associated with poorer outcomes, as expected. Sarcopenia was associated with poorer DFS and OS in the univariate analysis for both groups but remained significant in multivariate analysis only for patients treated by RT, likely due to a lack of statistical power in the RTCT group. This important finding indicates that, even with treatment with a very high rate of completeness (97%), such as RT, patients with sarcopenia have a poorer outcome.

Based on our results and recently published data [19], sarcopenia assessed by CT scans should be considered as an independent prognostic factor in future clinical trials. Nevertheless, the management of baseline sarcopenia seems to be very challenging. Due to the short delay available between HNSCC diagnosis and RT initiation, the correction of sarcopenia by muscle strengthening seems unlikely. Indeed, a randomized study recently showed that a 12-week intervention during RT in HNSCC patients did not reduce the loss of lean body mass, as assessed by a dual-energy X-ray absorptiometry scan [27].

This study had some limitations. First, the treatments varied from patient to patient, since the primary inclusion endpoint was the delivery of curative radiotherapy. As a re-sult, some patients received primary surgery and/or associated chemotherapy. Second, we used the gold-standard L3 CT scan to define LSM. However, L3 CT scans were not always available among HNSCC patients, and C3 CT scans were also currently investigated as a surrogate marker for LSM [14] and could help to standardize and diffuse the LSM definition in HNSCC. In this study, we lack data for patients treated with Erbitux [28]. Third, we were not able to determine weight loss during the weeks preceding inclusion in the study. Prediagnosis weight loss was more frequently observed than low BMI as an independent prognostic factor [29,30] and should be compared with sarcopenia in terms of prognostic value.

All inclusions were prospective with standardized treatment (radiotherapy, chemotherapy) and follow-up (cancer and nutritional follow-up). Patients were managed according to international recommendations for chemotherapy, IMRT radiotherapy, and supportive care. In addition, this was a large cohort with a gold-standard analysis of sarcopenia based on L3. Rigorous patient follow-up confirms the pejorative character of sarcopenia in these patients but also the absence of an impact on toxicity.

## 5. Conclusions

Considered together, our results suggest that, even among non-frail patients treated with curative intent with adequate nutritional support, sarcopenia evaluated by LSM identifies patients with poorer outcomes after treatment with either RT or RTCT.

## Figures and Tables

**Table 1:** Baseline characteristics of the patients. BMI: body mass index; WHO PS: World Health Organization performance status.

Characteristic	Total (*n* = 243)	Radiotherapy (*n* = 116)	Radiochemotherapy (*n* = 127)	*p*-Value
Sex ratio (M:F)	3.34	3.14	3.54	0.7
Age (years), mean ± SD	61.13 ± 9.04	63.41 ± 10.40	59.04 ± 6.99	0.00014
Age (median; Q1–Q3)	61 (56; 66)	62.5 (57; 70)	60 (54.5; 64)	
Smoking history, *n* (%)				0.25
Ever	198 (81.5)	98 (84.5)	100 (78.7)	
Never	45 (18.5)	18 (15.5)	27 (21.3)	
Chronic alcohol drinking, *n* (%)				0.86
Yes	118 (48.6)	57 (49.1)	61 (48.0)	
No	125 (51.4)	59 (50.9)	66 (52.0)	
BMI, mean ± SD	24.94 ± 4.56	24.78 ± 4.72	25.09 ± 4.43	0.59
Cancer site, *n* (%)				0.3
Oral cavity	69 (28.4)	39 (33.6)	30 (23.6)	
Oropharynx	79 (32.5)	32 (27.6)	47 (37.0)	
Hypopharynx	39 (16.0)	17 (14.7)	22 (17.3)	
Larynx	39 (16.0)	21 (18.1)	18 (14.2)	
Cancer of unknown primary (CUP)	17 (7.0)	7 (6.0)	10 (7.9)	
T stage, *n* (%)				0.0039
T0	17 (7.0)	7 (6.0)	10 (7.9)	
T1	45 (18.5)	29 (25.0)	16 (12.6)	
T2	79 (32.5)	45 (38.8)	34 (26.8)	
T3	51 (21.0)	18 (15.5)	33 (26.0)	
T4	51 (21.0)	17 (14.7)	34 (26.8)	
N stage, *n* (%)				<0.0001
N0	91 (37.4)	61 (52.6)	30 (23.6)	
N1	47 (19.3)	19 (16.4)	28 (22.0)	
N2	82 (33.7)	29 (25.0)	53 (41.7)	
N3	23 (9.5)	7 (6.0)	16 (12.6)	
Stage, *n* (%)				<0.0001
I	19 (7.8)	17 (14.7)	2 (1.6)	
II	50 (20.6)	30 (25.9)	20 (15.7)	
III	57 (23.5)	26 (22.4)	31 (24.4)	
IV	117 (48.1)	43 (37.1)	74 (58.3)	
p16 status (among oropharyngeal cancer patients), *n* (%)				0.09
Positive	23 (29.1)	5 (15.6)	18 (38.3)	
Negative	30 (38.0)	14 (43.8)	16 (34.0)	
Not known	26 (32.9)	13 (40.6)	13 (27.7)	
WHO PS, *n* (%)				0.15
0	97 (40.1)	41 (35.7)	56 (44.1)	
1	127 (52.5)	62 (53.9)	65 (51.2)	
2	18 (7.4)	12 (10.4)	6 (4.7)	
Undernutrition, *n* (%)				0.48
Yes	8 (3.3)	5 (4.4)	3 (2.4)	
No	231 (96.7)	108 (95.6)	123 (97.6)	
Sarcopenia, *n* (%)				0.12
Present	88 (36.7)	48 (41.7)	40 (32.0)	
Absent	152 (63.3)	67 (58.3)	85 (68.0)	
Performed surgery, *n* (%)				<0.0001
Yes	152 (62.6)	89 (76.7)	63 (49.6)	
No	91 (37.4)	27 (23.3)	64 (50.4)	

**Table 2:** Acute toxicities according to treatment and sarcopenia.

Toxicities	Radiotherapy (*n* = 116)						*p*-Value *	Radiochemotherapy (*n* = 127)						*p*-Value *
	Sarcopenia (*n* = 48)			No Sarcopenia (*n* = 67)				Sarcopenia (*n* = 40)			No Sarcopenia (*n* = 85)			
	Grade 0–1	Grade 2	Grade ≥ 3	Grade 0–1	Grade 2	Grade ≥3		Grade 0–1	Grade 2	Grade ≥ 3	Grade 0–1	Grade 2	Grade ≥ 3	
Mucositis	11 (22.9)	28 (58.3)	9 (18.8)	14 (20.9)	42 (62.7)	11 (16.4)	0.89	7 (17.5)	23 (57.5)	10 (25.0)	18 (21.4)	49 (58.3)	17 (20.2)	0.78
Dermatitis	16 (33.3)	28 (58.3)	4 (8.3)	27 (40.9)	33 (50.0)	6 (9.1)	0.69	13 (32.5)	23 (57.5)	4 (10.0)	34 (40.5)	42 (50.0)	8 (9.5)	0.66
Dysphagia	26 (56.5)	10 (21.7)	10 (21.7)	31 (47.7)	23 (35.4)	11 (16.9)	0.3	16 (40.0)	9 (22.5)	15 (37.5)	31 (36.9)	23 (27.4)	30 (35.7)	0.84
2 or 3 toxicities in one patient	42 (87.5)			59 (88.1)			0.93	35 (87.5)			70 (83.3)			0.55

* *p*-values compare sarcopenia vs. no sarcopenia.

**Table 3:** Univariate and multivariate analyses of disease-free survival (DFS) and overall survival (OS) for the entire population.

Characteristics	OS						DFS					
	Univariate			Multivariable			Univariate			Multivariable		
	HR	95% CI	*p*	HR	95% CI	*p*	HR	95% CI	*p*	HR	95% CI	*p*
Age	1.01	[0.98–1.04]	0.352				1.02	[0.99–1.04]	0.193			
BMI	0.95	[0.89–1]	0.068				0.97	[0.92–1.02]	0.182			
WHO PS 2 (vs. 0–1)	6.15	[3.12–12.11]	<0.001	4.26	[2.08–8.73]	<0.001	6.87	[3.84–12.3]	<0.001	4.14	[2.19–7.84]	<0.001
Stage III–IV (vs. I–II)	2.45	[1.24–4.84]	0.01	1.99	[0.96–4.12]	0.065	2.14	[1.22–3.76]	0.008	2.1	[1.11–3.94]	0.023
Tumor site												
Oropharynx	1											
CUP	0.64	[0.19–2.13]	0.463				0.76	[0.26–2.18]	0.605			
Oral cavity	0.92	[0.47–1.77]	0.795				1.36	[0.78–2.37]	0.282			
Hypopharynx	1.03	[0.47–2.25]	0.938				1.55	[0.82–2.96]	0.18			
Larynx	1.08	[0.53–2.18]	0.829				1.13	[0.59–2.18]	0.709			
Undernutrition	4.16	[1.51–11.5]	0.006				3.44	[1.39–8.52]	0.007			
Surgery	0.75	[0.45–1.25]	0.273				0.74	[0.48–1.15]	0.184	0.64	[0.37–1.09]	0.099
Sarcopenia	2.52	[1.51–4.20]	<0.001	1.9	[1.11–3.25]	0.019	2.45	[1.58–3.78]	<0.001	1.7	[1.06–2.71]	0.026
p16 Positivity	0.44	[0.14–1.39]	0.161				0.42	[0.15–1.16]	0.094			
RTCT	0.8	[0.49–1.33]	0.396				0.75	[0.48–1.15]	0.186	0.57	[0.34–0.96]	0.036

BMI: body mass index; WHO PS: World Health Organization performance status; CUP: cancer of unknown primary; RTCT: radiochemotherapy; HR: hazard ratio; CI: confidence interval.

**Table 4:** Univariate and multivariate analyses of disease-free survival and overall survival according to treatment (RT and RTCT).

Characteristics/Treatment	RT						RTCT					
	Univariate			Multivariable			Univariate			Multivariable		
PFS	HR	95% CI	*p*	HR	95% CI	*p*	HR	95% CI	*p*	HR	95% CI	*p*
Age	1.03	[1–1.06]	0.049				0.97	[0.93–1.02]	0.238	0.96	[0.91–1]	0.095
BMI	0.98	[0.92–1.05]	0.591				0.95	[0.88–1.03]	0.201			
WHO PS = 2	9.12	[4.20–19.8]	<0.001	5.9	[2.38–14.61]	<0.001	4.94	[1.91–12.79]	0.001	4.57	[1.74–11.98]	0.002
Stage III/IV (vs. I/II)	1.85	[0.99–3.49]	0.056	2.27	[1–5.14]	0.05	8.84	[1.21–64.41]	0.032	7.49	[1.02–55.13]	0.048
Tumor site												
Oropharynx							1					
CUP	1.31	[0.36–4.72]	0.679				0.33	[0.04–2.50]	0.283			
Oral cavity	1.09	[0.48–2.43]	0.843				1.72	[0.79–3.74]	0.169			
Hypopharynx	2.5	[1.03–6.08]	0.043				0.99	[0.38–2.60]	1			
Larynx	1.27	[0.52–3.06]	0.599				0.92	[0.33–2.55]	0.872			
Undernutrition	3.67	[1.13–11.9]	0.03	5	[1.37–18.32]	0.015	3.21	[0.77–13.39]	0.109			
Surgery	0.49	[0.26–0.94]	0.03	0.29	[0.13–0.62]	0.001	0.85	[0.45–1.61]	0.617			
Sarcopenia	2.94	[1.60–5.42]	<0.001	2.49	[1.26–4.9]	0.008	1.93	[1.02–3.67]	0.044			
P16 Positivity	1.02	[0.24–4.23]	0.98	5.04	[1.08–23.64]	0.04	0.29	[0.07–1.23]	0.093			
**OS**	**HR**	**95% CI**	***p***	**HR**	**95% CI**	***p***	**HR**	**95% CI**	***p***	**HR**	**95% CI**	***p***
Age	1.03	[0.99–1.07]	0.13				0.97	[0.92–1.03]	0.307	0.96	[0.90–1.02]	0.148
BMI	0.97	[0.89–1.05]	0.404				0.93	[0.85–1.02]	0.112			
WHO PS = 2	6.69	[2.73–16.4]	<0.001	4.23	[1.45–12.33]	0.008	5.15	[1.76–15.1]	0.003	4.84	[1.63–14.36]	0.004
Stage III/IV (vs. I/II)	2.12	[0.98–4.59]	0.057	2.69	[0.94–7.67]	0.064	6.58	[0.89–48.4]	0.064	5.59	[0.75–41.65]	0.092
Tumor site												
oropharynx	1						1					
CUP	1.33	[0.37–4.87]	0.664				0 *					
oral cavity	0.61	[0.23–1.61]	0.319				1.36	[0.55–3.35]	0.501			
hypopharynx	1.35	[0.46–3.99]	0.585				0.8	[0.26–2.48]	0.695			
larynx	1.03	[0.39–2.72]	0.947				1.06	[0.37–3.01]	0.914			
Undernutrition	6.24	[1.88–20.7]	0.003	12.25	[2.93–51.15]	<0.001	2.18	[0.29–16.1]	0.445			
Surgery	0.6	[0.28–1.31]	0.2	0.4	[0.15–1.04]	0.06	0.75	[0.36–1.57]	0.448			
Sarcopenia	2.71	[1.31–5.59]	0.007	2.24	[1.03–4.86]	0.041	2.21	[1.06–4.59]	0.034			
P16 Positive	0.71	[0.10–5.27]	0.742				0.39	[0.09–1.64]	0.198			

BMI: body mass index; WHO PS: World Health Organization performance status; CUP: cancer of unknown primary; RTCT: radiochemotherapy; HR: hazard ratio; CI: confidence interval. * no event observed

## Data Availability

Data are available from the Clinical Research Unit, Henri Becquerel Center, 76038 Rouen, France.

## Supplementary Materials

The following are available online at https://www.mdpi.com/2072-6694/13/4/753/s1: Table S1: baseline characteristics of the patients according to treatment (RT or RTCT) and sarcopenia status.
